# PCAF Involvement in Lamin A/C-HDAC2 Interplay during the Early Phase of Muscle Differentiation

**DOI:** 10.3390/cells9071735

**Published:** 2020-07-20

**Authors:** Spartaco Santi, Vittoria Cenni, Cristina Capanni, Giovanna Lattanzi, Elisabetta Mattioli

**Affiliations:** 1 Unit of Bologna, CNR Institute of Molecular Genetics “Luigi Luca Cavalli Sforza”, 40136 Bologna, Italy; spartaco.santi@cnr.it (S.S.); vittoria.cenni@cnr.it (V.C.); ccapanni@area.bo.cnr.it (C.C.); 2 IRCCS Istituto Ortopedico Rizzoli, 40136 Bologna, Italy

**Keywords:** PCAF, lamin A/C, HDAC2, Emery–Dreifuss muscular dystrophy type 2, muscle differentiation

## Abstract

Lamin A/C has been implicated in the epigenetic regulation of muscle gene expression through dynamic interaction with chromatin domains and epigenetic enzymes. We previously showed that lamin A/C interacts with histone deacetylase 2 (HDAC2). In this study, we deepened the relevance and regulation of lamin A/C-HDAC2 interaction in human muscle cells. We present evidence that HDAC2 binding to lamin A/C is related to HDAC2 acetylation on lysine 75 and expression of p300-CBP associated factor (PCAF), an acetyltransferase known to acetylate HDAC2. Our findings show that lamin A and farnesylated prelamin A promote PCAF recruitment to the nuclear lamina and lamin A/C binding in human myoblasts committed to myogenic differentiation, while protein interaction is decreased in differentiating myotubes. Interestingly, PCAF translocation to the nuclear envelope, as well as lamin A/C-PCAF interaction, are reduced by transient expression of lamin A mutated forms causing Emery Dreifuss muscular dystrophy. Consistent with this observation, lamin A/C interaction with both PCAF and HDAC2 is significantly reduced in Emery–Dreifuss muscular dystrophy myoblasts. Overall, these results support the view that, by recruiting PCAF and HDAC2 in a molecular platform, lamin A/C might contribute to regulate their epigenetic activity required in the early phase of muscle differentiation.

## 1. Introduction

A fine regulation of acetyltransferases (HAT) and histone deacetylases (HDACs) activity is at the basis of all physiological cellular processes including skeletal myogenesis [1,2]. It has been demonstrated that activation of epigenetic enzymes and the consequent affinity with different partners depend on their ability to self-regulate or regulate each other through post-translational modifications [3]. In particular, HDAC2 function is regulated through its acetylation, a modification that, in cardiomyocytes, is finely balanced by acetyltransferase p300-CBP associated factor (PCAF) and HDAC5 activity [4]. Interestingly, HDAC2 and PCAF are involved in the early phase of muscle differentiation by coordinating MyoD acetylation and its transactivation activity [5,6]. In cycling myoblasts, HDAC2 deacetylates and represses MyoD. A protein complex containing MyoD and HDAC2, was in fact observed at transcriptionally inactive muscle regulatory DNA regions in myoblasts [5,7,8]. However, when myoblasts exit from the cell cycle, PCAF replaces HDAC2 and acetylates MyoD, inducing muscle gene transcription [6,9].

Lamin A/C is the major constituent of the nuclear lamina. Mature lamin A is produced from its precursor prelamin A by complex post-translational processing including farnesylation, double endoprotease cleavage and carboxymethylation [10]. First, full-length prelamin A is subjected to farnesylation on the cysteine of the CaaX motif, subsequently the aaX tripeptide is cleaved by ZMPSTE24 or RCE1 (Ras-converting enzyme 1) and the C-terminal cysteine residue is carboxymethylated by ICMT (isoprenylcysteine carboxymethyltransferase). Finally, proteolytic removal of the farnesylated C-terminus end by a specific endoprotease (ZMPSTE24) leads to mature lamin A [11]. Lamin A/C has been demonstrated to play a key role during myoblast differentiation [12,13,14]. In particular, lamin A/C association with the regulatory regions of muscle genes is able to influence muscle differentiation [15]. However, prelamin A has also been shown to regulate early events of myoblast differentiation, as caveolin 3 expression [16] and nuclear positioning in human muscle cells [17]. We also demonstrated that lamin A and prelamin A are complexed to Ankrd2, a protein involved in the transcriptional response to stress during myoblast differentiation [18,19]. *LMNA* gene mutations are responsible for the onset of autosomal dominant Emery–Dreifuss muscular dystrophy (EDMD2), which is characterized by skeletal muscle weakness and wasting, joint contractures and cardiomyopathy [18,19]. A correlation between epigenetic changes at the myogenic loci and *LMNA* mutations causing EDMD2, has been reported [20,21]. Moreover, EDMD2 cells showed altered activity of muscle-specific transcription factors including MyoD [22,23].

We have previously demonstrated that lamin A/C binds HDAC2 in human fibroblasts [24]. While lamin A/C-HDAC2 interaction is not affected by the HDAC2 phosphorylation state, HDAC2 acetylation might regulate its binding to A type lamins, as suggested by the observation that HDAC inhibitors known to favor HDAC2 acetylation [4] were able to increase lamin A/C-HDAC2 binding [25]. Based on these considerations, we evaluated a potential involvement of acetyltransferases in the regulation of lamin A/C-HDAC2 complexes and identified the acetyltransferase PCAF as a modulator of HDAC2 binding to lamin A/C. Moreover, we observed that lamin A was able to recruit PCAF to the nuclear lamina and recruitment was increased in the presence of farnesylated prelamin A.

A main role of lamin A/C interplay with epigenetic enzymes in myogenesis has been demonstrated by several studies [26,27]. Lamin A/C is able to interact with epigenetic repressors that control muscle differentiation, regulating self-renewal of muscle satellite stem cells [26,28]. Interestingly, emerin, a binding partner of lamin A/C, is able to regulate epigenetic activity of HDAC3 during muscle differentiation, and a perturbation of histone acetylation state was observed in emerin-deficient myogenic progenitors [29]. Here, we demonstrated that lamin A/C interaction with both PCAF and HDAC2 are modulated in murine and human myoblasts during myogenic differentiation. Of note, we found that PCAF targeting to the nuclear lamina was reduced by *LMNA* mutations causing EDMD2 and lamin A/C-HDAC2 interaction was impaired in EDMD2 myoblasts. These results add to the evidence that a functional lamina is required for epigenetic enzyme regulation during myogenesis.

## 2. Materials and Methods

### 2.1. Cell Culture, Differentiation and Transfection

HEK293 cells (human embryonic kidney cells) were cultured in Dulbecco’s modified Eagle’s medium, supplemented with 10% fetal bovine serum (FBS), and were transfected with HA-PCAF or FLAG-tagged plasmids containing: wild-type prelamin A, LA-WT, which undergoes normal maturation; LA-C661M, which cannot be farnesylated; LA-L647R, which is farnesylated but cannot undergo endoproteolysis. *LMNA* pathogenetic mutants used in this study were: LA-R527P and LA-R401C, linked to EDMD2 [18]. HA tagged PCAF in pCI expression vector was a gift from Dr. Melanie Ott, Gladstone Institute, UCSF, San Francisco, CA (USA). Flag tagged HDAC2 in pCMV10–3xFlag vector has been previously described [24,30]. Flag-HDAC2 construct was used as a template for QuickChange site-directed mutagenesis (Agilent Technologies, Milan, Italy) with primers carrying the indicated mutations. Primers used for mutagenesis were purchased from IDT technologies (Tema Ricerca, Milan, Italy) and were as follows:

mHDAC2-K75R-F: 5’-AGCGATGAGTATATCAGGTTTCTACGATCAATA-3’
mHDAC2 K75R-R: 5’-TATTGATCGTAGAAACCTGATATACTCATCGCT-3’
mHDCA2-K75Q-F: 5’-AGCGATGAGTATATCCAGTTTCTACGATCAATA-3’
mHDCA2-K75Q-R: 5’-TATTGATCGTAGAAACTGGATATACTCATCGCT-3’

After mutagenesis, ORF of each plasmid was verified by a DNA sequencing service (BMR, Padua, Italy).

Transfection of HEK293 cells was performed using Fugene 6 solution (Promega, San Luis, CA, USA) according to the manufacturer’s instructions and cells were incubated for 24 h after transfection. C2C12 cells (mouse myoblast cell line) were cultured in Dulbecco’s modified Eagle’s medium, supplemented with 10% FBS and differentiated with 2% of horse serum (HS) for 12 h to obtain committed myoblasts and 24 h to obtain early stage myotubes. Human myoblast cultures from control donor and EDMD2 patients Y259D-*LMNA* (Patient1) and L140P-*LMNA* (Patient2), were obtained from the BioLaM biobank (approved by the “Rizzoli Orthopedic Institute Ethics Committee” on 05/09/2016. Prot. gen “0018250-01-13”). All EU and local ethical rules were respected. Cell cultures established as reported [17] were cultured in Dulbecco’s modified Eagle’s medium (DMEM), supplemented with 20% fetal bovine serum (FBS) (Gibco Life Technologies, Milan, Italy) and antibiotic-anti-mycotic solution (Sigma, St. Louis, MO, USA).

### 2.2. Antibodies and Drugs

Antibodies employed were: anti-PCAF (sc-13124) from Santa Cruz (Dallas, TX, USA) used 1:50 for immunofluorescence (IF) or 1:100 for western blotting (WB); anti-lamin A/C, goat polyclonal (SC-6215) from Santa Cruz (Dallas, TX, USA) used at 1:100 dilution for and in situ proximity ligation assay (PLA); anti-HDAC2, rabbit polyclonal (AB16032) from Abcam (Cambridge, UK) used at 1:2000 for WB and 1:200 for PLA analysis; anti-H3K9 acetylated, rabbit polyclonal (07-352) from Merck Millipore (Milan, Italy) used at 1:200 for IF; anti-FLAG tag (F3165) from Sigma (St.Louis, MO, USA) and anti-HA tag (sc-7392) from Santa Cruz (Dallas, TX, USA) were used at 1:1000 in WB and 1:300 in IF; anti-GFP (sc-9996) from Santa Cruz (Dallas, TX, USA) was used at 1:500 in WB; anti acetyl-lysine (9824S) from Cell Signaling (Leiden, The Netherlands) was used at 1:2000 in WB; anti myogenin from Santa Cruz (Dallas, TX, USA) was used at 1:100 in IF.

A total of 2 μM of Mevinolin (Sigma, St. Louis, MO, USA), a drug able to induce non-farnesylated prelamin A accumulation through inhibition of farnesyl production, was added to HEK293 cells for 18 h. A total of 100 μM of Sirtinol, a SIRT1 inhibitor, able to induce PCAF acetylation [31], was added to human fibroblasts for 18 h; while 50 μM of sirtuin 1 activators (MC 2562 and MC 2528) PCAF deacetylating drugs were added for 18 h.

### 2.3. In Situ Proximity Ligation Assay

In situ proximity ligation assay (PLA) was performed using kits from Sigma (St. Louis, MO, USA): Duolink^®^ In Situ Detection Reagents Orange (DUO92007) according to manufacturer instructions. Briefly, methanol-fixed samples were treated with 4% BSA in PBS to saturate non-specific binding and were incubated with primary antibodies overnight at 4 °C. Thereafter, slides were incubated for 1 h at 37 °C with secondary probes diluted to final concentrations of 1:5. Ligation solution was added to each sample and slides were incubated in a humidity chamber for 30 min at 37 °C. Later, ligation solution was removed with wash buffer A and amplification solution was added to each sample. Slides were incubated in a humidity chamber for 100 min at 37 °C and then washed with wash buffers. Duolink in situ mounting medium with DAPI was added to the slides and samples were observed by a Nikon Eclipse Ni fluorescence microscope equipped with a digital CCD camera and NIS Elements AR 4.3 software. Quantitative analysis of PLA results was performed using Duolink Image Tool software (Sigma, St. Louis, MO, USA) by counting 200 nuclei per sample.

### 2.4. Co-Immunoprecipitation Experiments

Transfected HEK293 cells were subjected to IP lysis buffer containing: 50mM Tris-HCl (ph = 8), 150 mM NaCl; 1% NP-40; 1.5 mM MgCl; 1 mM DTT; 1 mM PMSF; 20 mM NaF; 0.05% SDS; phosphatases and proteases inhibitors. After sonication, clarification and protein quantification, 700 μg of lysate for samples were immunoprecipitated with 1 μL/mL of anti-FLAG antibody (Sigma, St.Luis, MO, USA) over-night at 4 °C. After the addition of 30 µL/mL of protein A/G (Santa Cruz, Dallas, TX, USA) for 60 min at 4 °C, the immunoprecipitated proteins were washed with lysis buffer 3 times. Subsequently, immunoprecipitated samples were added to Laemmli’s buffer and subjected to immunoblotting analysis. Incubation with primary and secondary antibodies was performed and immunoblotted bands were revealed by Invitrogen ECL detection system. Densitometric analysis was performed with ImageJ2 program.

### 2.5. Immunofluorescence Analysis

Cells grown on coverslips were fixed with absolute methanol at room temperature for 10 min or with 4% paraformaldehyde for 15 min at 4 °C (plus 5 min with 0.02% triton x-100 for permeabilization). After saturation of non-specific binding with PBS, containing 4% BSA for 20 min, coverslips were incubated with primary antibodies overnight at 4 °C or 1 h, and revealed with FITC, Cy5 or TRIC-conjugated secondary antibodies diluted at 1:100 (incubated for 1 h at RT). Samples were mounted with an anti-fade reagent (Molecular Probes Life Technologies, Milan, Italy) and observed with different microscopes.

### 2.6. Imaging

Immunofluorescence and PLA analysis were performed using a Nikon Eclipse Ni epifluorescence microscope with 40× and 100× objectives. The images captured with NIS-Elements 4.3 AR software, were elaborated using Photoshop CS.

Confocal analysis was performed using a Nikon A1 confocal laser scanning microscope, equipped with a 60×, 1.4 NA objective and with 405, 488, and 561 nm laser lines. Z-stacks were collected at optical resolution of 80 nm/pixel, stored at 12-bit with 4096 different gray levels, pinhole diameter set to 1 Airy unit and z-step size to 200 nm.

Nikon-Structured Illumination Microscopy (3D N-SIM) which permits observing fluorescent samples at resolutions below the limit the diffraction of light imposed by optical microscopy (85–100 nm) was performed using a Plan-Apochromat 100×/1.49 Oil TIRF objective and 488 and 561 nm laser lines. For each axial plane of a 3D stack 1024 × 1024 pixel images and 4096 gray levels were acquired in 3 rotations and 5 different phases. Final images (recorded at z-step size of 125 nm) were reconstructed using NIS Elements Advanced Research software (Nikon, Tokyo, Japan). Protein colocalization was evaluated by comparing the equivalent pixel positions of green and red signals of fluorophores in each of the acquired images (optical sections). A two-dimensional scatter plot diagram of the individual pixels from the paired images was generated and a threshold level of signal to be included in the analysis was selected. Pixels with intensity values greater than 50% grey levels (on a scale from 0 to 4096) were selected for both signals, and the colocalization binary maps that indicate regions containing highly colocalized signals were imaged and merged (in white) to the green and red signals [32,33]. Image analysis (feature measurements, 3D object count and 3D rendering) was performed using NIS-Elements Advanced Research software (Nikon, Tokyo, Japan).

## 3. Results

### 3.1. PCAF Promotes HDAC2-Lamin A/C Interaction

**Figure 1:**
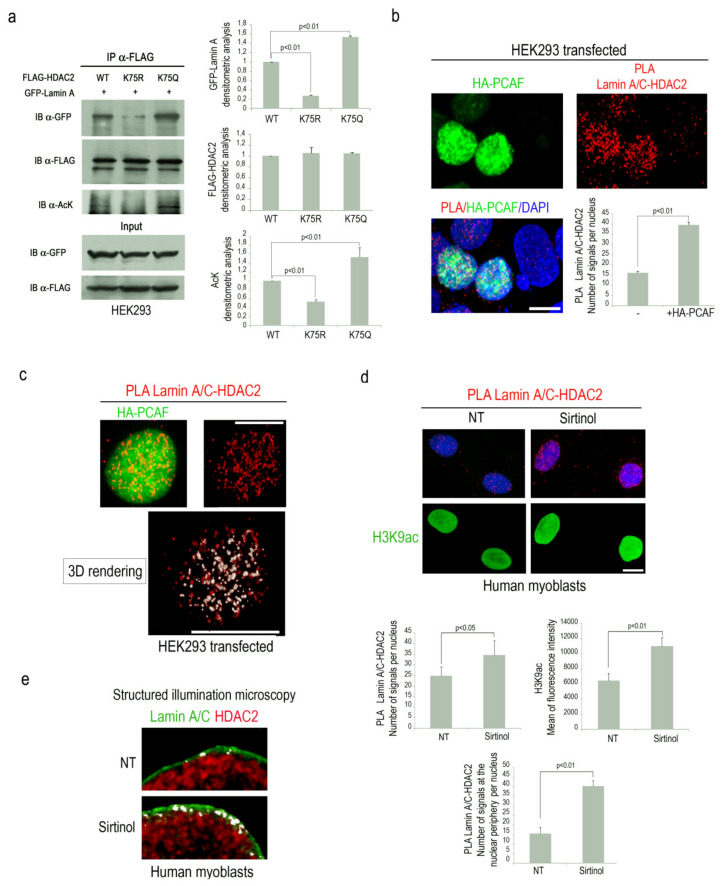
p300-CBP associated factor (PCAF) strengthens lamin A/C-HDAC2 interaction. (**a**) Co-immunoprecipitation of GFP-lamin A with wild-type or mutant FLAG-HDAC2 in HEK293 cells; immunoblotting (IB) of immunoprecipitated samples (IP) revealed for GFP-lamin A, FLAG-HDAC2 and acteyl-lysine (AcK); Input, whole lysate of samples used for IP. Densitometric analysis of immunoblotted bands is reported in the graphs. (**b**) HEK293 cells subjected to immuno-fluorescence staining for HA-PCAF (green) and lamin A/C-HDAC2 proximity ligation assay (PLA) (red dots). (**c**) Immunostaining of HA-PCAF (green) and PLA of lamin A/C and HDAC2 (red dots). A rendered 3D analysis of lamin A/C-HDAC2 PLA signals (red dots) and colocalization with HA-PCAF (white signals) is shown (3D rendering). (**d**) Lamin A/C-HDAC2 PLA (red dots) and immunofluorescence staining of H3K9ac (green) in human myoblasts left untreated (NT) or treated with sirtinol (Sirtinol). (**e**) Structured illumination microscopy analysis of lamin A/C (green) and HDAC2 (red) in untreated (NT) or sirtinol-treated human myoblasts (Sirtinol). White signals are generated by colocalization of lamin A/C and HDAC2. Quantitative analysis of PLA signals is reported in the graphs in **b** and **d**; in panels **b** and **d**, 4,6-diamidino-2-phenylindole (DAPI, blue) was used to counterstain cell nuclei. Scale bars, 10 µm. At least three biological replicates were used in each experiment. Statistically significant differences between values (*p* < 0.05 or *p* < 0.01) are indicated.

Our recent findings supported the hypothesis that HDAC2 acetylation could influence lamin A/C-HDAC2 interplay [25]. To test this hypothesis, co-immunoprecipitation experiments were performed in HEK293 overexpressing GFP-lamin A and FLAG-HDAC2 acetylation mutants. Interestingly, a lower amount of GFP-lamin A was immunoprecipitated by acetylation defective HDAC2, compared to wild type HDAC2 (Figure 1a). On the contrary, an increased amount of immunoprecipitated GFP-lamin A was revealed in samples overexpressing acetylation mimetic HDAC2-K75Q (Figure 1a). Acetylation state of HDAC2 was confirmed by an anti-acetyl lysine specific antibody (Figure 1a). These results demonstrated that HDAC2 acetylation on lysine 75 promotes its binding to lamin A/C.

Since it has been demonstrated that the acetyltransferase PCAF induces HDAC2 acetylation on lysine 75 [4], we decided to investigate PCAF involvement in the formation of lamin A/C-HDAC2 complexes. To this end, we performed a proximity ligation assay (PLA) to test the effect of PCAF overexpression on the interaction between lamin A/C and HDAC2. PLA quantitative analysis demonstrated that lamin A/C-HDAC2 binding was significantly increased in HEK293 cells overexpressing PCAF (Figure 1b). Interestingly, in this condition, high levels of colocalization between PCAF and lamin A/C-HDAC2 complexes were observed by confocal miscroscopy analysis (Figure 1c, white signal). A rendered 3D analysis revealed that most of lamin A/C-HDAC2 complexes colocalized with PCAF (Figure 1c and Video S1).

To further support the evidence that PCAF activity might influence HDAC2 affinity for lamin A/C, human myoblasts were treated with sirtinol, a SIRT1 inhibitor able to induce PCAF autoacetylation and activation [31,34]. Higher lamin A/C-HDAC2 interaction was detected by PLA in sirtinol-treated cells and acetylation of H3K9, a well-known PCAF substrate, was also increased (Figure 1d) [35]. On the contrary, PCAF deacetylation by two different sirtuin 1 activators (MC 2825 or MC 2562) led to a reduction of lamin A/C-HDAC2 binding (Figure S1). Interestingly, colocalization analysis of lamin A/C and HDAC2 immunolabeling by Structured Illumination Microscopy (SIM) showed an increased number of “contact areas” (white signals) in sirtinol-treated human myoblasts as compared to untreated cells (Figure 1e). These results demonstrated that PCAF activity enhances HDAC2 binding to lamin A/C, suggesting the existence of a molecular complex at the nuclear periphery consisting of lamin A/C, HDAC2 and PCAF.

### 3.2. PCAF-Lamin A/C Interaction During the Early Phase of Muscle Differentiation

**Figure 2:**
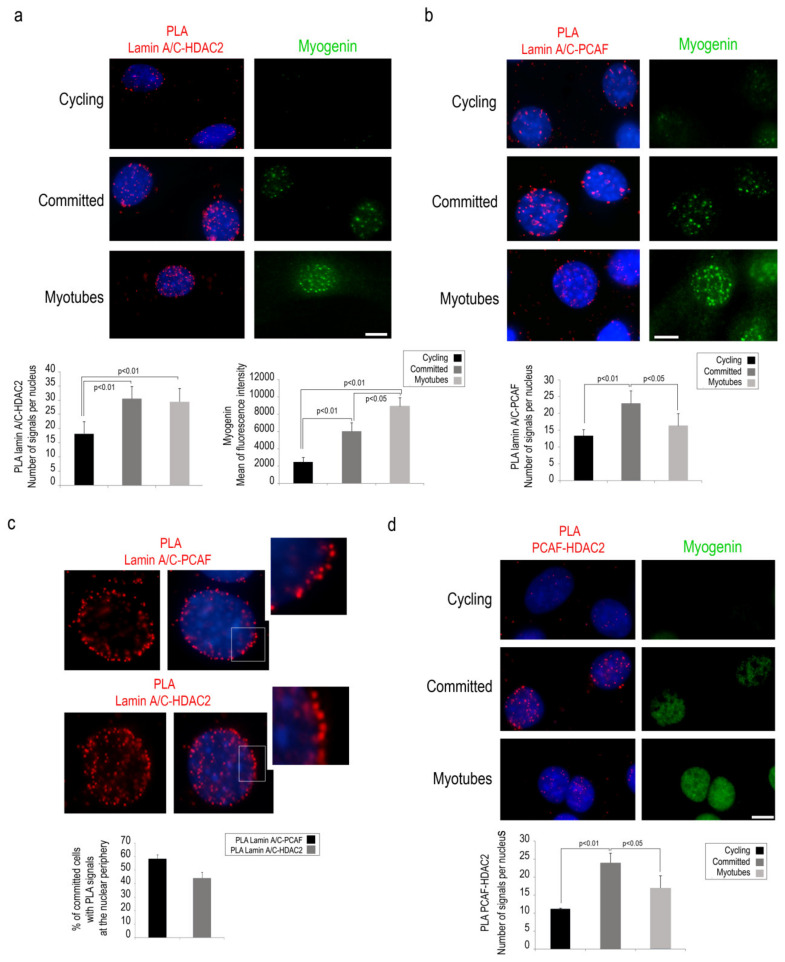
Lamin A/C binds PCAF and HDAC2 in committed C2C12 cells. (**a**) Lamin A/C- HDAC2 PLA (red dots) in cycling and committed myoblasts or myotubes. Myogenin (green) was used as a marker of myogenic differentiation. (**b**) PCAF-lamin A/C PLA (red signal) in cycling myoblasts, committed myoblast and myotubes. (**c**) Lamin A/C-PCAF and lamin A/C -HDAC2 PLA (red dots) in representative myoblasts. Inset, high magnification of the area indicated by a rectangle. (**d**) PCAF-HDAC2 PLA (red dots) and immunofluorescence staining of myogenin (green) in cycling or committed myoblasts and in myotubes. Quantitative analysis of PLA signals is reported in the graphs. DAPI (blue staining) was used to counterstain cell nuclei. Scale bars, 10 µm. Three biological replicates were used in each experiment. Statistically significant differences between values (*p* < 0.05 or *p* < 0.01) are indicated.

During muscle differentiation, PCAF and HDAC2 have opposite regulatory effects and the balancing between these enzymes regulates MyoD acetylation and its transactivation activity [5,6]. For these reasons, we decided to investigate a possible involvement of lamin A/C in the formation of the enzyme platform consisting of PCAF and HDAC2 during the early phase of muscle differentiation. First, PLA was performed to detect lamin A/C interaction with PCAF or HDAC2 in cycling C2C12 myoblasts or in myoblasts committed to differentiation. HDAC2 binding to lamin A/C was slightly detected in cycling cells, whereas a strongly increased PLA signal was observed in committed myoblasts and persisted in myotubes (Figure 2a). Also, PCAF-lamin A/C PLA signals were detected at the highest level in committed myoblasts while they were significantly decreased in myotubes (Figure 2b).

Interestingly, enrichment at the nuclear periphery of lamin A/C-PCAF and lamin A/C-HDAC2 complexes was observed in committed cells (Figure 2c). Moreover, PCAF interaction with HDAC2 was increased in committed C2C12 myoblasts (Figure 2d). These results strongly suggest the presence of a dynamic complex including PCAF, Lamin A/C and HDAC2 at the nuclear envelope of committed myoblasts.

### 3.3. Lamin A Recruits PCAF to the Nuclear Lamina

**Figure 3:**
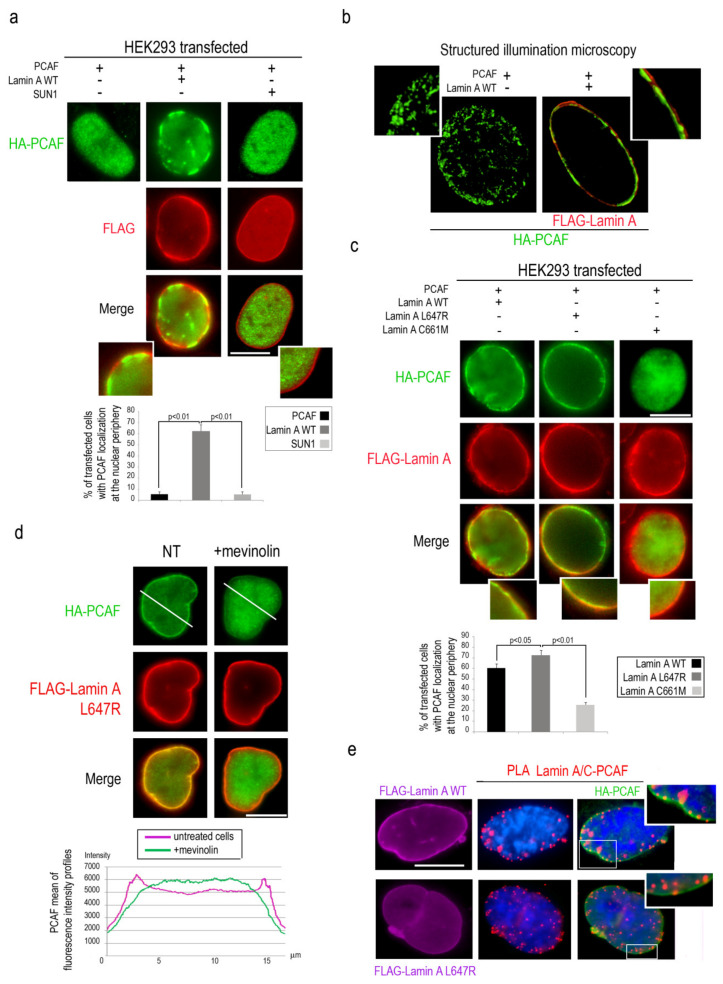
Lamin A recruits and binds PCAF at the nuclear lamina. (**a**) HEK293 co-transfected with HA-PCAF (green), FLAG-lamin A-WT (red), FLAG-SUN1 (red) subjected to immunostaining. High magnification of merged images at the bottom of the panel. % of transfected cells with PCAF localization at the nuclear envelope was reported in the graph. (**b**) Structured illumination microscopy of FLAG-lamin A-WT (red) and HA-PCAF (green) in co-transfected HEK293 cells. (**c**) Immunofluorescence analysis of HEK293 cells co-transfected with HA-PCAF (green) and wild-type FLAG tagged lamin A, lamin A L647R or lamin A C661M (red signal). High magnification of merged images at the bottom of the panel. % of transfected cells with PCAF localization at the nuclear envelope was reported in the graph. (**d**) HA-PCAF (green) and FLAG lamin A-L647R (red) immunostaining in untreated (NT) or mevinolin-treated HEK293 cells (+mevinolin). Mean of fluorescence intensity profiles of PCAF in untreated (violet) or mevinolin-treated cells (green) is shown. A measure of intensity profiles of 50 transfected nuclei was performed by NIS Elements AR 4.3 software and a mean of intensity profile was shown in the graph. (**e**) PCAF-lamin A/C PLA (red dots) and PCAF immunofluorescence staining (green) in HEK293 cells expressing HA-PCAF and FLAG-lamin A WT or FLAG-lamin A L647R (violet). DAPI (blue) was used to counterstain cell nuclei. Inset, high magnification of the area indicated by a rectangle. Scale bars, 10 µm. Three biological replicates were used in each experiment. Statistically significant differences between values (*p* < 0.05 or *p* < 0.01) are indicated.

To deepen the relationship between PCAF and lamin A/C, HEK293 cells were co-transfected with HA-tagged PCAF and FLAG-Lamin A and immunofluorescence analysis was performed with antibodies against the respective tags. In cells overexpressing PCAF alone, PCAF had a typical nucleoplasmic localization [36], while co-expression of PCAF with lamin A triggered PCAF localization at the nuclear lamina in a high percentage of cells (Figure 3a). As a negative control, SUN1 co-expression with PCAF did not change PCAF nucleoplasmic localization (Figure 3a). SIM analysis of several planes passing through the center of the nucleus further demonstrated PCAF recruitment to the nuclear lamina in cells overexpressing lamin A (Figure 3b).

It has been reported that lamin A processing intermediates are accumulated in myoblasts during myogenic differentiation [16,17]. In particular, farnesylated prelamin A is accumulated in human myoblasts and persists in mature muscle [17]. To test whether farnesylated prelamin A might differently modulate PCAF translocation to the nuclear lamina, HEK293 cells were transfected with HA-PCAF and FLAG-lamin A mutants encoding for lamin A processing intermediates and subjected to immunostaining. Overexpression of LA-L647R, causing accumulation of farnesylated prelamin A, induced PCAF recruitment to the nuclear lamina more efficiently than overexpressed LA-WT (Figure 3c). On the other hand, when PCAF was co-expressed with LA-C661M (eliciting accumulation of non-farnesylated prelamin A), the percentage of cells with PCAF recruitment to the nuclear periphery was strongly reduced (Figure 3c). In agreement with the latter finding, PCAF localization to the nuclear periphery in cells overexpressing LA-L647R, was strongly reduced by treatment with mevinolin (Figure 3d), a drug able to impair prelamin A farnesylation thus inducing accumulation of non-farnesylated prelamin A [25]. Fluorescence intensity profiles for the green channel confirm the different distribution of PCAF in treated and untreated cells (Figure 3d). Interestingly, PLA analysis showed the existence of an interaction between these proteins in HEK293 cells overexpressing PCAF and wild-type or LA-L647R lamin A (Figure 3e). Immunofluorescence analysis confirmed PCAF nuclear lamina localization and colocalization with PLA signals (Figure 3e, high magnification). These findings demonstrated that PCAF recruitment to the nuclear lamina is directed by mature lamin A as well as by farnesylated prelamin A.

### 3.4. PCAF-Lamin A/C Interaction is Altered by LMNA Mutations Causing EDMD Phenotype

**Figure 4:**
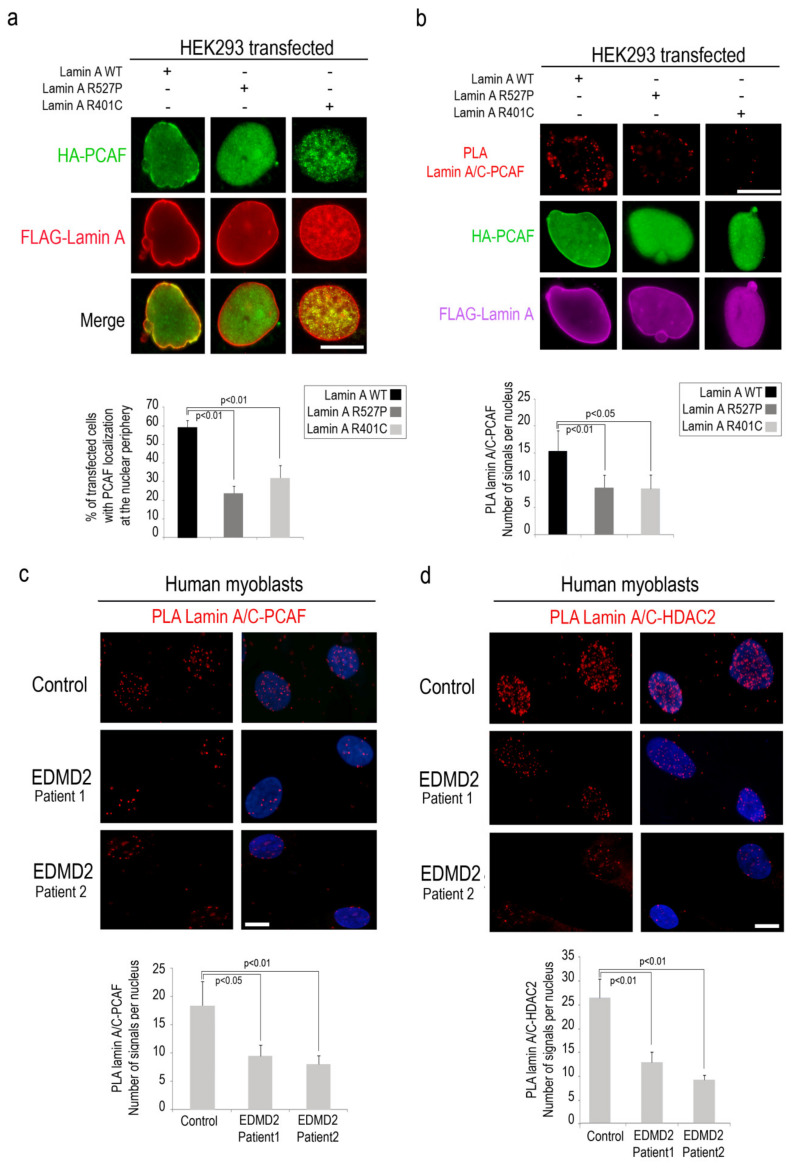
Altered lamin A/C-PCAF interaction is determined by mutated lamin A. (**a**) Immunofluorescence analysis of HEK293 cells co-transfected with HA-PCAF (green) and FLAG tagged wild-type lamin A, lamin A-R527P or lamin A-R401C (red signal). % of transfected cells with PCAF localization at the nuclear envelope was reported in the graph. (**b**) PLA between PCAF and lamin A/C (red dots) in HEK293 cells co-transfected with HA-PCAF (green) and FLAG Lamin A-WT or EDMD2 mutants (violet). (**c**) Human myoblasts from control (control) and EDMD2 patients (EDMD2 Patient1 and EDMD2 Patient2) subjected to lamin A/C-PCAF PLA (red dots). (**d**) Human myoblasts from control (control) and EDMD2 patients (EDMD2 Patient1 and EDMD2 Patient2) subjected to lamin A/C-HDAC2 PLA (red dots). Quantitative analysis of PLA signals is reported in the graphs in panels **b**, **c** and **d**. DAPI (blue) was used to counterstain cell nuclei in panels **c** and **d**. Scale bars, 10 µm. Three biological replicates were used in each experiment. Statistically significant differences between values (*p* < 0.05 or *p* < 0.01) are indicated.

Then, we wondered if EDMD2-linked *LMNA* mutations could affect PCAF recruitment to the nuclear lamina and enzymatic activity. To test this possibility, we performed immunofluorescence analysis of HEK293 cells co-expressing HA-PCAF and EDMD2-linked LA-R527P and LA-R401C. In cells expressing lamin A pathogenetic mutants, we observed a reduced percentage of nuclei with PCAF recruitment to the nuclear envelope (relative to cells expressing wild-type lamin A) (Figure 4a). Interestingly, in the same samples, we observed a reduced interaction between mutated lamin A and PCAF with respect to cells expressing lamin A wild-type by PLA (Figure 4b). To support the evidence that PCAF affinity for lamin A/C is altered by EDMD2-linked *LMNA* mutations, we performed PLA in EDMD2 myoblasts committed to myogenic differentiation. In agreement with the data reported above, significant decrease of lamin A/C-PCAF interaction was observed in EDMD2 myoblasts relative to controls, supporting the hypothesis that intact lamin A/C is necessary for PCAF interaction in muscle cells (Figure 4c and Figure S2).

Since we observed that HDAC2 acetylation by PCAF influences its binding to lamin A/C, we decided to investigate HDAC2-lamin A/C interaction in EDMD2 cells. Interestingly, PLA demonstrated a reduction of lamin A/C-HDAC2 binding in EDMD2 cells with respect to controls, both in committed myoblasts and myotubes (Figure 4d and Figure S3). These results suggest that PCAF modulates lamin A/C-HDAC2 binding during muscle differentiation.

## 4. Discussion

Recent literature demonstrates an emerging role of lamin A/C in epigenetic regulation of myogenesis [20,26]. We previously showed that lamin A/C is able to bind HDAC2 and regulate its activity during stress response [24]. Here, we demonstrate that lamin A/C-HDAC2 interaction is promoted by PCAF-dependent HDAC2 acetylation on lysine 75. This result is consistent with previous findings showing that HDAC inhibitors able to induce HDAC2 acetylation, such as Trichostatin A [4], increase HDAC2-lamin A/C interaction [25]. In this study, we further show lamin A/C-PCAF interaction in committed myoblasts and modulation of protein binding during muscle differentiation, an event affecting lamin A/C-HDAC2 interplay. Our results show the formation of a dynamic complex including lamin A/C, PCAF and HDAC2 in the early phase of muscle differentiation. At this stage, PCAF is known to accumulate at the nuclear level to induce growth arrest and myogenic differentiation [37,38].

**Figure 5:**
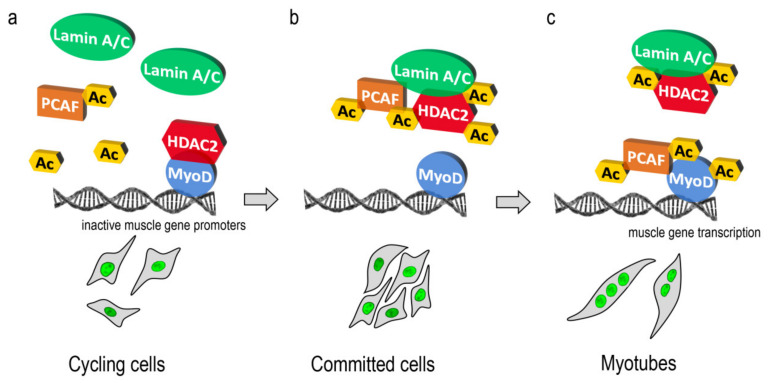
Cartoon representing a working hypothesis. (**a**) In cycling myoblasts, lamin A/C (green) is located near both PCAF (orange) and HDAC2 (red); at the same time HDAC2 binds MyoD (blue) to repress muscle gene promoters. (**b**) In committed myoblasts lamin A/C recruits PCAF to permit HDAC2 acetylation (Ac yellow square) and displacement from MyoD. In this phase, a dynamic complex including lamin A/C-PCAF and HDAC2 could be present. (**c**) Finally, in myotubes, PCAF might be released from lamin A/C complexes and recruited to MyoD to activate the transcription factor through acetylation.

Our findings allow us to speculate that lamin A/C could contribute to HDAC2-PCAF dynamic interaction aimed at the regulation of muscle-specific transcription factors. For instance, during myogenesis, an antagonistic function of PCAF and HDAC2 on MyoD activation has been demonstrated [5,6,9]. In cycling myoblasts, HDAC2 directly interacts with MyoD keeping it in a deacetylated and inactive state and suppressing muscle-specific gene expression [5,7], whereas HDAC2-MyoD binding decreases in differentiating cells, when active PCAF replaces HDAC2 to acetylate MyoD contributing to the muscle differentiation program [5,6]. Here, we demonstrate that lamin A/C favors PCAF-HDAC2 interplay in committed myoblasts, an event that increases HDAC2 acetylation and lamin A/C binding and could promote PCAF-MyoD interaction. In support of this possibility, it has been reported that pan-HDAC inhibition, which increases lamin A/C-HDAC2 interaction [25], induces MyoD acetylation improving dystrophic muscle regeneration [39,40]. Our findings suggest a working hypothesis that might explain the dynamic involvement of lamin A/C in epigenetic enzyme regulation during muscle differentiation (Figure 5).

Interestingly, we observe that PCAF recruitment to the nuclear lamina is significantly increased by farnesylated prelamin A, the lamin A precursor, which is accumulated during differentiation of human muscle cells [16,17]. While our studies showed a role of farnesylated prelamin A in nuclear positioning in human muscle [17], here we provide evidence of prelamin A involvement in epigenetic enzyme regulation, which is consistent with multiple regulatory functions of the lamin A precursor during myogenesis.

In this context, it is possible that lamin A/C-HDAC2 complexes, in differentiating muscle cells could also serve to move HDAC2 away from transcriptionally inactive loci of muscle genes, thus promoting gene expression. In support of this hypothesis, it has been demonstrated that lamin A/C binding to muscle gene promoters was reduced when myoblasts were induced to differentiate, while histone deacetylase inhibitors, reduced lamin A/C localization to the myogenic promoters [15].

The altered lamin A/C interaction with HDAC2 and PCAF in EDMD2 myoblasts suggests that this function is also relevant to disease pathogenesis. Despite our previous results that showed that lamin A/C-HDAC2 interaction is not affected in human fibroblasts carrying pathogenetic EDMD2 mutations [24], here we found a reduced lamin A/C-HDAC2 binding in EDMD2 myoblasts and myotubes. These findings suggest that, while the altered lamin A/C-HDAC2 interplay in EDMD2 myoblasts is related to defective lamin A/C-PCAF interaction, lamin A/C binding to HDAC2 in EDMD2 fibroblasts could involve other regulatory proteins or pathways.

The extent to which *LMNA* mutations affect lamin A/C-PCAF binding and PCAF or HDAC2 recruitment might differently impair the expression of different muscle-specific genes. In this perspective, aberrant epigenetic enzyme regulation by mutated lamin A/C could contribute to the phenotypic variability observed in laminopathic patients. Finally, given the role of lamin A/C in epigenetic enzyme regulation, epigenetic drugs could be a new challenge for a therapeutic approach to laminopathies. For instance, modulating lamin A/C-HDAC2 binding by TSA in EDMD2 could be tested as an approach that might improve the transcriptional activity of muscle cells through improved PCAF-dependent HDAC2 acetylation.

## Supplementary Materials

The following are available online at https://www.mdpi.com/2073-4409/9/7/1735/s1, **Video S1**. High levels of colocalization between PCAF and lamin A/C-HDAC2 complexes were observed by confocal microscopy in a rendered 3D analysis. **Figure S1**. Decrease of lamin A/C-HDAC2 binding in cells treated with Sirt1 activators. **Figure S2**. A reduced lamin A/C interaction with PCAF was observed in EDMD2 human myoblasts. **Figure S3**. A reduced lamin A/C interaction with both PCAF and HDAC2 was observed in EDMD2 human myoblasts and myotubes.
